# Multi release software reliability modelling incorporating fault generation in detection process and fault dependency with change point in correction process

**DOI:** 10.1038/s41598-025-04102-4

**Published:** 2025-07-02

**Authors:** Sujit Kumar Pradhan, Anil Kumar, Vijay Kumar

**Affiliations:** 1 Department of Mathematics, Birla Institute of Technology and Science, Pilani, K K Birla Goa Campus, Zuarinagar, Sancoale, Goa 403726 India; 2 Department of Mathematics, Birla Institute of Technology and Science, Pilani, K K Birla Goa Campus, Zuarinagar, Sancoale, Goa 403726 India; 3 Department of Mathematics, Amity Institute of Applied Sciences, Amity University, Noida, 201313 Uttar Pradesh India

**Keywords:** Non-homogeneous Poisson process, Fault detection process, Fault correction process, Software reliability growth models, Mean value function., Software, Computer science, Computational science

## Abstract

Many software reliability growth models (SRGMs) have been proposed in the literature to evaluate the remaining faults and software reliability. The probability of getting failure-free software within a specified period and environment is known as software reliability and is recognized as one of the essential aspects. In this paper, we present a new SRGM of the fault detection process (FDP) and fault correction process (FCP) and study the dependency between the FDP and FCP as the amount of fault dependency, not time dependency. The FDP is modeled by considering a multi-release concept where the leftover faults from the previous release and newly added faults after enhancing the existing features in the software are considered. Further, the FCP model is proposed by introducing the change point concept in the fault dependency function. These models are validated on two actual medium-sized software system data sets. The results show that the proposed models fit the data set more accurately than the existing SRGMs. We have also discussed the optimal release time through a cost model where setup, testing, and debugging costs are introduced in both the testing and operational phases in the cost model.

## Introduction

The utilization of computer systems has grown extensively in various sectors, such as industry, banking, security, health, etc. Therefore, the dependence on hardware and software systems has rapidly increased. Since software systems are embedded in our daily lives, the performance of a software system is essential. The motivation behind the SRGMs is to describe the behaviour of software failure during the testing process and define it as the probability of failure-free software for a specified period within a specified environment.

Research in software and hardware reliability has been done over the past five decades. The software’s reliability also determines the software testing and optimal release times. Several researchers^1–11^ have developed SRGMs using the non-homogeneous Poisson process (NHPP) to assess and predict software reliability. These models incorporate various factors such as fault detection^1,3^, correction processes^4,9^, effort allocation^2,5,7,11^ and environmental uncertainties^6,8,10^ to better reflect real-world software testing scenarios. Apart from this, other real-world issues such as real-time monitoring, fault diagnosis, and similar challenges can also be explored in the context of practical implications^12–14^. The contributions of these studies have significantly improved reliability estimation techniques, aiding in more efficient software development and release planning. Many existing SRGMs^1,15^ have been developed assuming that a fault is immediately corrected whenever a fault is detected. A real issue like imperfect debugging has been considered and incorporated in SRGMs^4,16,17^. Various information is obtained from SRGMs during the software development process, such as cost analysis^17–19^, testing resources allocation^11,20^, and software release time^21–23^.

The software tester team tests the software to detect the fault. During the software testing process, each detected fault is diagnosed and corrected. It is not easy to fix a detected fault immediately; some time is required to fix the fault, known as a debugging delay. The time lag between fault detection and correction is addressed by Schneidewind^24^ and introduced the idea of a fault correction process (FCP) followed by a fault detection process (FDP) by introducing random time delay, which follows an exponential distribution. Later on, Xie et al.^25^ provided FDP and FCP by taking different time delays such as constant, time-dependent, and random. Wu et al.^26^ presented an FCP model by taking random time delays such as exponential, normal distributed, and gamma time delays. Huang et al.^27^ incorporated fault dependency and debugging time delay in the software reliability growth model.

Lo et al.^28^ presented an SRGM model where the fault detection and correction process is integrated. Peng et al.^29^ incorporated imperfect debugging in a testing effort-dependent FDP and FCP model. Jia et al.^30^ discussed an SRGM where FCP follows the Markovian process. Chatterjee et al.^31^ discussed FDP and FCP in which they incorporate the fault reduction factor and the fault reduction factor, which follows the Weibull curve. Saraf et al.^32^ proposed a general framework for modeling fault detection and fault correction process where imperfect debugging, change point, and fault generation are considered. Pradhan et al.^33^ presented an SRGM discussing the change point in the testing effort rate function. The FDP and FCP models discussed above were developed as single-release software reliability growth models. Much research has not been done on the multi-release FDP and FCP model. Yang et al.^34^ proposed an approach for modeling single and multi-release fault detection and correction processes with time delays following gamma distribution.

Although all the FDP and FCP models discussed above can effectively evaluate software reliability , there are some disadvantages to these models, which motivated us to propose FDP and FCP in a new way. A time delay function used in the FDP model is developed by assuming the delay in correction time^25,26,34,35^. This is not the case because the derivation of time delay functions did not use the testing data, which may not be a realistic concept to distinguish the relationship between the FDP and FCP. Another thing is that the time delay functions, which follow random distributions, may not give a better result regarding the model’s parameter estimation and performance. However, the time dependency depends upon various factors like the ability of the tester team, the complex nature of the detected faults, resource availability, etc. Till now, no FDP model has been proposed through the multi-release concept. Similarly, the change-point concept has not been discussed in any FCP model. Therefore, we are motivated to propose the dependency between the FDP and FCP through the number of faults, where the multi-release and change point strategy concepts are also incorporated in the FDP and FCP model, respectively. This is a new way to propose FDP and FCP models incorporating multi-release and change-point ideas.

In this work, we have proposed the fault detection process (FDP) and fault correction process (FCP) model in a new setup. The FCP model is discussed through fault detection by taking the amount of fault dependency rather than time dependency. The fault detection process model is proposed through a multi-release concept by extending the ideas of Zhu and Pham^36^. This paper considers two types of faults: the remaining fault from the previous release and the new faults generated from the current release while modeling FDP. Further, we have assumed that the remaining faults of the previous release and the new ones from the current release are categorized as types I and II. Type I software fault is defined as an independent and easy-detected fault, and type II is defined as a dependent and difficult-detected fault^37^. After fault detection, the software tester team fixes those faults. During the fault correction process, the same testing strategies do not work for different types of faults to remove them. Therefore, different testing strategies are used to fix those faults. The point of time where the change in the testing strategies is observed can be termed as a strategic point or change point. Therefore, we have incorporated the change point, where the software manager can change strategy during the fault correction. The essence of this study is captured in the following points:

- The multi-release concept has been introduced in the FDP model.
- The FCP model has been proposed using the fault dependency function.
- The change point concept associated with the fault dependency function in the FCP model has been introduced.
- The optimal software release time has also been discussed.

The remaining part of the paper is structured as follows: Section "Methodology and model formulation" comprise notations used throughout the paper, model assumptions, fault detection and correction process, and their relationship. Section "Numerical and data analysis"  discusses software failure data, change-point calculation, parameter estimation, model comparison criteria and software reliability. The model’s parameters are estimated using the least squares method on two real data sets related to the software failure. The proposed and selected models’ predictive performances are based on the goodness-of-fit criteria. The optimal software release time is discussed in section "Optimal software release policy". Section “Conclusion”  illustrates the conclusion and several potential directions for future research of the paper.

## Methodology and model formulation

We begin by describing the development process of the proposed SRGM, highlighting its fundamental assumptions and mathematical formulation to model the fault detection and correction processes. To avail of failure-free and reliable software, software testing is required. The software testing process follows a NHPP, and the cumulative number of detected fault *N*(*t*) is expressed as follows:

1 $$\begin{aligned} P\{N(t)=n\}=\frac{{m(t)}^n}{n!}\text {exp}(-m(t)),\; \text {for}\; n=0,1,2, \ldots . \end{aligned}$$

The effect of NHPP assumptions on this study’s analysis and prediction should be explicitly stated, as these assumptions play a crucial role in shaping the fault detection process, reliability estimation, and overall model performance. The NHPP-based models assume that software failures occur according to a time-dependent fault detection rate, which impacts how faults accumulate and how efficiently debugging is performed. Furthermore, NHPP influences the mean value function (MVF), which estimates the cumulative number of software failures detected over time, thereby affecting the accuracy of reliability predictions. The mean value function *m*(*t*), i.e., the expected number of detected faults, is represented in terms of intensity function $\lambda (t)$ as

2 $$\begin{aligned} m(t)=\int _{0}^{t} \lambda (z)dz. \end{aligned}$$

**Fig. 1:**
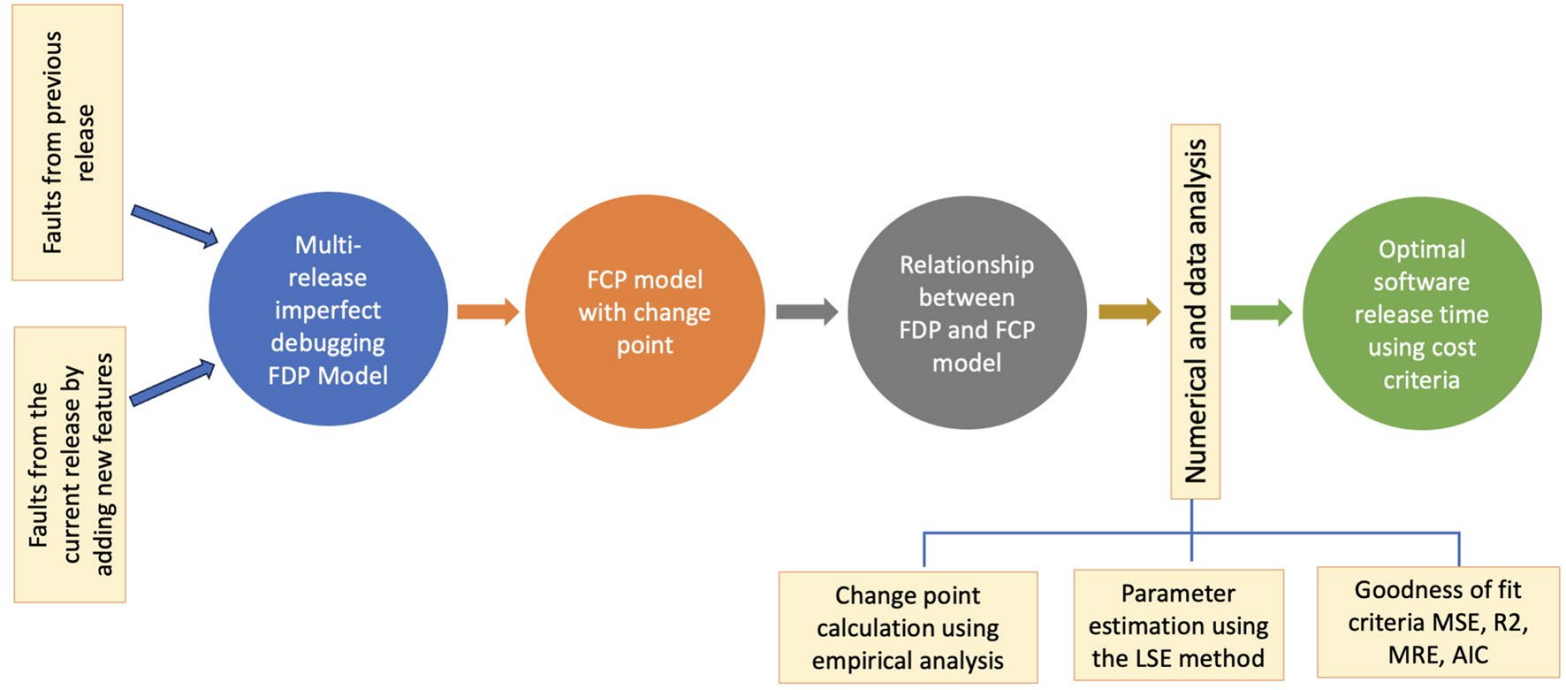
Structure of proposed model.

Figure 1 illustrates the various steps involved in the model formulation, providing a clear understanding of the proposed approach. It visually represents the key components and their interconnections, offering insight into the overall structure of the proposed model.

### Notations

The study employs several symbols and abbreviations essential for understanding the proposed model, all of which are compiled in Table 1 for reference.

**Table 1:** Notations.

$m_d(t)$	The number of faults detected at time *t*.
$m_c(t)$	The number of faults corrected at time *t*.
*p*(*t*)	The number of software faults from the previous release.
*q*(*t*)	The number of software faults in the current release after adding new features.
$\alpha$	The rate at which new software faults are added in FDP.
*b*(*t*)	The fault detection rate function.
*r*(*t*)	The fault dependency function.
$\tau$	The strategic point or change-point.
$t_{lc}$	The length of the software life cycle.

### Model assumptions

The basic assumptions are taken to formulate the proposed models as follows:

1. The fault detection in SRGM follows the non-homogeneous Poisson process.
2. The mean number of detected faults at any time *t* is proportional to the number of remaining faults from the previous release and new faults from the current release after adding new features.
3. All the detected faults from the previous release and new ones from the current release are categorized as types I and II, respectively.
4. After detecting software faults, a debugging process occurs immediately.
5. New faults can be introduced into the software during debugging due to imperfect debugging.
6. Although the debugging process starts without any delay, the detected faults cannot be corrected immediately, and the dependency between fault detection and correction processes is represented by *r*(*t*), i.e.,
  3 $$\begin{aligned} r(t) = \frac{m_c(t)}{m_d(t)}. \end{aligned}$$
7. The fault dependency function *r*(*t*) may be changed at some time $\tau$, which is referred to as a change-point.

### Fault detection process (FDP) model

Generally, a software product is released to the market with basic features according to the market demand. A software system has a finite lifetime due to the need for new software or because, over time, its maintenance cost exceeds its initial price. Therefore, the software system is withdrawn from the market for a new release. Much research^4,17,38^ and references therein has been done on a single version of the software system. Modeling and predicting software failure are also carried out through a single version of the software system. Zhu and Pham^36^ have incorporated the multi-release concept in open-source software by introducing a dependent fault detection rate. In the multi-release SRGM, they have considered (*i*) the remaining faults from the previous release and (*ii*) the new faults from the current release after adding new features. We have extended the concept proposed by Zhu and Pham^36^ to propose our FDP model. Thus, the FDP model, together with the dependent fault detection rate, is as follows.

4 $$\begin{aligned} \frac{dm_d(t)}{dt} = b(t)\left( p(t)-m_d(t)\right) \left( q(t)-m_d(t)\right) ,\; m_d(0)=0. \end{aligned}$$

Correcting all the faults during the debugging phase of a single-release software is difficult due to time constraints and resource availability. So, the software tester team always tries to correct a maximum number of faults; thus, in the software’s first release, some faults remain in it. Therefore, when the software goes for the next release, some faults that are not removed during the previous release are carried forward. Hence, we have considered that the total number of faults from the previous release is *p*(*t*). When new features are added to enhance existing features in the previous software, it is always necessary to ensure that new features will be consistent with previous ones. New features are developed as a single module and integrated with the previously existing ones, called previous modules. During the development time of the new module, the fault *q*(*t*) is the sum of *q* (initial number of faults) and $\alpha m_d(t)$ (new faults added due to integration testing). When the new module is integrated with the earlier modules, they interact, and as a result, other new faults are added with a rate of $\alpha$ in the current release^39^. We have assumed the total number of faults from the previous release is constant and new faults of the current release are as follows.

5 $$\begin{aligned} p(t)=p \;\; \text {and}\; \; q(t)=q+\alpha m_d(t). \end{aligned}$$

The fault detection rate shows an *S*-shaped varying trend. Therefore, it is enough to describe fault detection using a *S*-shaped curve. In our model, the fault detection process depends on the remaining faults from the previous and new faults from the current release. We have addressed the fault detection rate *b*(*t*) proposed by Yamada et al.^3^ and used by several researchers^40,41^ in Eq. (6) by assuming that it is a learning process as

6 $$\begin{aligned} b(t)=\frac{b^2\, t}{1+bt},\;b>0. \end{aligned}$$

where *b* is the asymptotic unit of software fault detection rate. On solving Eq. (4) using Eqs. (5) and (6), we get the following mean value function for the fault detection process

7 $$\begin{aligned} m_d(t)=\frac{pq\left( (1+bt)^p-e^{-(q+p(\alpha -1)bt)}(1+bt)^{q+p\alpha }\right) }{q(1+bt)^p-p(1-\alpha )e^{-(q+p(\alpha -1)bt)}(1+bt)^{q+p\alpha }}. \end{aligned}$$

### Fault correction process (FCP) model with change point

The fault correction process is modeled based on the assumptions taken. Usually, a fault is corrected after the detection of faults in FDP. When the software tester team performs testing, easy and simple faults are detected first. Therefore, the number of detected faults grows very fast. But at the same time, the software tester team needed to become more familiar with the software. As a result, fewer faults are corrected than detected faults. As software testing increases, the software tester team gets more information about the software, and the number of corrected faults increases due to the learning process. At the same time, the number of detected faults decreases as time increases, and the difference between FDP and FCP decreases. At the end of testing, it is difficult to find new faults, and almost all detected faults are corrected. Therefore, the fault correction process lags far behind the fault detection process at the initial testing phase, i.e., the ratio of corrected faults to the number of detected faults. Hence, in line with Li and Pham^42^ and Shu et al.^43^, we represent the number of corrected faults from Eq. (3) as

8 $$\begin{aligned} m_c(t)=m_d(t)r(t),\;\;m_c(0)=0. \end{aligned}$$

During the FDP, researchers assumed that the failure occurred due to the independent and random faults, following the same distribution^44^. The software failure distribution affects the testing environment, resource allocation, and testing strategy. When the software tester team changes the resource allocation and testing strategy, a change-point occurs in a model^45–47^. As per assumptions, we have considered that the detected faults are of two types, i.e., type I and type II. Usually, type I faults are known as simple ones that can be removed easily, and type II faults aren’t easily known as hard ones. The software tester team adopts two testing effort strategies to remove these two types of faults. As a result, the fault correction rates are different. When the software tester team experienced a different fault correction rate, the ratio of corrected faults to the number of detected faults also differed. Therefore, we have considered the change-point concept in the fault dependency function *r*(*t*). Thus, the time when the software tester team changes the testing effort strategies is called the change point, denoted by $\tau$. Hence, the following FCP model from Eq. (8) with a change point is as follows.

9 $$\begin{aligned} m_c(t)= {\left\{ \begin{array}{ll} m_d(t)r_1(t), & 0\le t \le \tau ,\\ m_d(t)r_2(t), & t > \tau . \end{array}\right. } \end{aligned}$$

Since the ratio of the number of faults corrected to the number of faults detected phenomenon also follows an *S*-shaped curve, we have considered two different *S*-shaped functions, say $r_1(t)=\frac{1}{1+\beta e^{-r t}}$ and $r_2(t)=\frac{1}{1+\gamma e^{-\sigma t}}$, and hence, the FCP model (9), using (7) is reduced to the following

10 $$\begin{aligned} m_c(t)= {\left\{ \begin{array}{ll} \frac{pq\left( (1+bt)^p-e^{-(q+p(\alpha -1)bt)}(1+bt)^{q+p\alpha }\right) }{q(1+bt)^p-p(1-\alpha )e^{-(q+p(\alpha -1)bt)}(1+bt)^{q+p\alpha }}\left( \frac{1}{1+\beta e^{-r t}}\right) , & 0\le t \le \tau ,\\ \frac{pq\left( (1+bt)^p-e^{-(q+p(\alpha -1)bt)}(1+bt)^{q+p\alpha }\right) }{q(1+bt)^p-p(1-\alpha )e^{-(q+p(\alpha -1)bt)}(1+bt)^{q+p\alpha }}\left( \frac{1}{1+\gamma e^{-\sigma t}}\right) , & t > \tau . \end{array}\right. } \end{aligned}$$

The mean value function for the existing and proposed models are listed in Table 2, in which 9 paired FDP and FCP models are listed. Out of 9 paired FDP and FCP models, model numbers 1 to 8 are existing models, and model number 9 is the proposed model. The paired FDP and FCP model performances are evaluated from data sets I (DSI) and II (DSII).

**Table 2:** Summary of existing models and proposed models.

Model no	Model name	MVF
1	FDP: G-O model^1^ FCP: G-O model with constant time delay^25^	$m_d(t)=a(1-e^{-bt})$ $m_c(t)=a(1-e^{-b(t-c)})$
2	FDP: G-O model FCP: G-O model with time-dependent delay^25^	$m_d(t)=a(1-e^{-bt})$ $m_c(t)=a(1-(1+ct)e^{-bt})$
3	FDP: G-O model FCP: G-O model with exponential distributed delay^25^	$m_d(t)=a(1-e^{-bt})$ $m_c(t)=a(1-\frac{c}{c-b}e^{-bt}+\frac{b}{c-b}e^{-ct})$
4	FDP: G-O model FCP: G-O model with normally distributed time delay^26^	$m_d(t)=a(1-e^{-bt})$ $m_c(t)=-ae^{-bt+\mu b+b^2 {\sigma }^2/2}\big (\phi (t,b{\sigma }^2+\mu ,\sigma )-\phi (0,b{\sigma }^2+\mu ,\sigma )\big )+a(\phi (t,\mu ,\sigma )+\phi (0,\mu ,\sigma ))$
5	FDP: G-O model FCP: G-O model with gamma distributed time delay^25^	$m_d(t)=a(1-e^{-bt})$ $m_c(t)=a\Gamma (t,\alpha ,\beta )-\frac{ae^{-bt}}{(1-b\beta )^\alpha }\Gamma (t,\alpha ,\frac{\beta }{1-b\beta })$
6	FDP: Li et al.^42^ FCP: Li et al.^42^	$m_d(t)=\frac{a}{1-\alpha }\bigg (1-e^{-c(1-\alpha )t^r}\bigg )$ $m_c(t)=\frac{a}{1-\alpha }\bigg (1-e^{-c(1-\alpha )t^r}\bigg )\frac{1}{1+be^{-\beta t}}$
7	FDP: Li et al.^42^ FCP: Li et al.^42^	$m_d(t)=\frac{a}{1-\alpha }\bigg (1-(1+rt)^{1-\alpha }e^{-r(1-\alpha )t}\bigg )$ $m_c(t)=\frac{a}{1-\alpha }\bigg (1-(1+rt)^{1-\alpha }e^{-r(1-\alpha )t}\bigg )\frac{1}{1+be^{-\beta t}}$
8	FDP: Li et al.^42^ FCP: Li et al.^42^	$m_d(t)=\frac{a}{1-\alpha }\Bigg (1-\bigg (\frac{(1+c)e^{-rt}}{1+ce^{-rt}}\bigg )^{1-\alpha }\Bigg )$ $m_c(t)=\frac{a}{1-\alpha }\Bigg (1-\bigg (\frac{(1+c)e^{-rt}}{1+ce^{-rt}}\bigg )^{1-\alpha }\Bigg )\frac{1}{1+be^{-\beta t}}$
9	Proposed FDP model Proposed FCP model	$m_d(t)=\frac{pq\Big \{(1+bt)^p-e^{-(q+p(\alpha -1)bt)}(1+bt)^{q+p\alpha }\Big \}}{q(1+bt)^p-p(1-\alpha )e^{-(q+p(\alpha -1)bt)}(1+bt)^{q+p\alpha }}$ $$m_c(t)= {\left\{ \begin{array}{ll} m_d(t)\Big (\frac{1}{1+\beta e^{-r t}}\Big ), & 0\le t \le \tau \\ m_d(t)\Big (\frac{1}{1+\gamma e^{-\sigma t}}\Big ), & t > \tau \end{array}\right. }$$

### Relationship between FDP and FCP Model

**Fig. 2:**
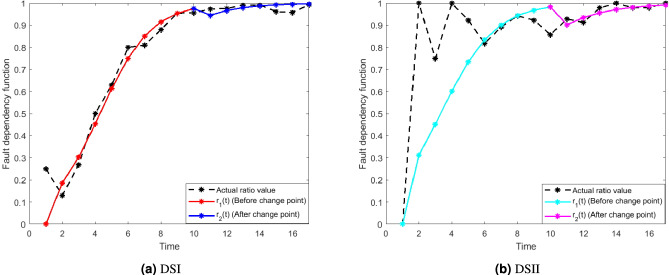
The fault dependency function for DSI and DSII.

In this subsection, the relationship between the FDP and FCP model has been studied in terms of the fault dependency function *r*(*t*). From model assumption 6, the fault dependency function is the ratio of the number of faults corrected to the number of faults detected. Numerous type I faults are initially detected rapidly, but due to testers’ unfamiliarity with the software, fault correction lags behind detection. It results in a low ratio of corrected and detected faults, which may decrease over time. As testing continues, testers gain experience, leading to faster fault removal and an increasing proportion of corrected faults over time. However, as faults become more complex, the growth in detected faults slows down, and the gap between fault correction and detection may diminish. Towards the end of testing, the number of detected faults stabilizes, and the ratio of corrected to detected faults approaches 1 as all faults are nearly corrected. Therefore, the range of the fault dependency function (*r*(*t*)) lies between 0 and 1. We have taken two different *S*-shaped fault dependency functions, i.e., $r_1(t)=\frac{1}{1+\beta e^{-r t}}$ and $r_2(t)=\frac{1}{1+\gamma e^{-\sigma t}}$ before and after the change point respectively. Two failure data sets (DSI & DSII) are collected to validate the fault dependency functions. Figure 2 illustrates the relationship between the FDP and FCP model, with the vertical axis representing the fault dependency function and the horizontal axis representing time.

## Numerical and data analysis

### Software failure data

In this subsection, the proposed model’s parameters estimation and performance have been done using two software failure data sets. The first data set (DSI) is collected from testing a medium-size software system^26^ and shown in Table 3. The testing process took 17 weeks, and during the process, a total of 144 and 143 faults were detected and corrected, respectively. In Table 4, the second data set (DSII)^31^ is presented. During the 17 weeks of the testing process, the cumulative number of detected and corrected faults is 54.

**Table 3:** Data set I (Failure data from medium size software system^26^).

Weeks	Actual number of detected faults	Actual number of corrected faults	Weeks	Actual number of detected faults	Actual number of corrected faults
1	12	3	10	114	109
2	23	3	11	116	113
3	43	12	12	123	120
4	64	32	13	126	125
5	84	53	14	128	127
6	97	78	15	132	127
7	109	89	16	141	135
8	111	98	17	144	143
9	112	107			

**Table 4:** Data set II^31^.

Weeks	Actual number of detected faults	Actual number of corrected faults	Weeks	Actual number of detected faults	Actual number of corrected faults
1	1	0	10	42	36
2	2	2	11	42	39
3	4	3	12	46	42
4	5	5	13	47	46
5	13	12	14	47	47
6	22	18	15	49	48
7	28	25	16	51	50
8	35	33	17	54	54
9	39	36			

### Change-point

Usually, the software tester team knows the change point while testing software. However, for the given two datasets, the change point is unknown; therefore, to find the change-point for the datasets, we have used the software failure increasing rate concept^37^, which is given as follows

11 $$\begin{aligned} y'(t)=\lim _{\delta t \rightarrow 0} \frac{y(t+\delta t)-y(t)}{\delta t}, \end{aligned}$$

where $y'(t)$ is the fault correction rate, *y*(*t*) is observed cumulative number of corrected faults by time *t*, and $y(t+\delta t)$ is observed cumulative number of corrected faults by time $t+\delta t$.

**Fig. 3:**
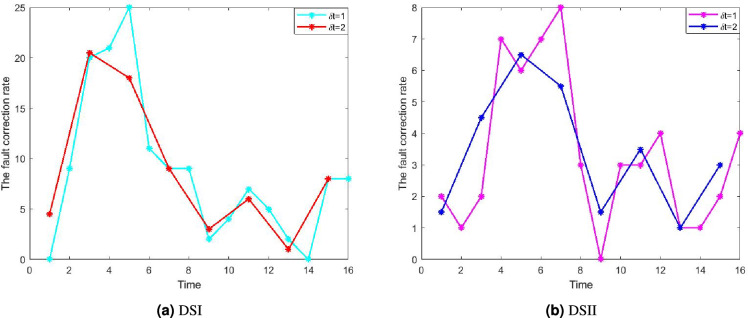
The fault correction rate for different values of $\delta t$.

Figure 3a shows the fault correction rate for different $\delta t$ values for DSI. The figure shows that the fault correction rate has two peaks at $t=5$ and $t=15$. Type II faults follow the correction of type I faults and need different testing efforts. Initially, the software tester team had less knowledge about the faults and wasn’t familiar with them. After some time, they are familiar with the faults and have more information about them. As a result, the fault correction rate increases and attains its maximum, and after some time, it starts decreasing to reach its stable position. The decreasing nature of the fault correction rate happens due to the presence of a smaller number of type I faults. After that, the tester team applies different testing effort strategies to correct the type II faults, and a similar pattern is found, like type I faults. Hence, the point at which the tester team changes the testing effort strategy is taken as a strategic point or change point ($\tau$). Therefore, we have concluded from Fig. 3a that the change-point for DSI is $\tau =10$. The fault correction rate for different $\delta t$ values for DSII is depicted in Fig. 3b. Applying a similar argument to DSII, we considered the change-point $\tau =10$.

### Parameter estimation

To estimate the model’s parameter for the paired model of fault detection $m_d(t)$ and correction $m_c(t)$, nonlinear least square estimation (LSE) is used, in which the parameters are estimated by minimizing the sum of squared residuals, i.e., the difference between the mean value function and the observed data set for both the detection and correction process as

12 $$\begin{aligned} \sum _{i=1}^{n}\left[ (m_d(t_i)-y_{d_i})^2 + (m_c(t_i)-y_{c_i})^2\right] , \end{aligned}$$

where $m_d(t_i)$ and $m_c(t_i)$ are the estimated cumulative number of detected and corrected faults, and $y_{d_i}$ and $y_{c_i}$ are the cumulative number of detected and corrected faults at time $t_i$, respectively.

### Criteria for model comparison

In this subsection, the comparative analysis of the paired SRGM (FDP and FCP) is discussed. The standard criteria for comparing the models are the mean-squared error (MSE), $R^2$, and mean relative error (MRE). The smaller value of MSE and MRE and the maximum value of $R^2$ give a better software reliability model.

The MSE measures the average of the deviation between the predicted values and the actual data in both the fault detection and correction process, which is defined as^42^

$$\begin{aligned} MSE=\frac{1}{2n} \sum _{i=1}^{n}\left[ (m_d(t_i)-y_{d_i})^2 + (m_c(t_i)-y_{c_i})^2\right] , \end{aligned}$$

where *n* is the number of observations in the model. Generally, the coefficient of determination is defined as^48^

$$\begin{aligned} R^2=1-\frac{SS_{res}}{SS_{tot}}, \end{aligned}$$

where $$\displaystyle SS_{res}=\sum _{i=1}^{n} \left( m(t_i)-y_i\right) ^2, \;\; SS_{tot} = \sum _{i=1}^{n} \left( m(t_i)-{\bar{y}}\right) ^2 \;\; \text{ and } \;\; {\bar{y}} = \frac{1}{n} \sum _{i=1}^{n} y_i.$$

The mean coefficient of determination $R^2$ for both fault detection and correction process is given as

$$\begin{aligned} R^2=\frac{R_d^2+R_c^2}{2}, \end{aligned}$$

where $R_d^2$ and $R_c^2$ are the coefficients of determination for fault detection and correction process, respectively. The capability of a model is predicted by calculating the mean relative errors (MRE) from the predicted faults and observed faults as^26^

$$\begin{aligned} \text {MRE}=\frac{1}{n}\sum _{i=1}^{n}\bigg |\frac{m(t_i) - y_i}{y_i} \bigg |, \end{aligned}$$

where $m(t_i)$ and $y_i$ are the predict fault and the observed fault at time $t_i$, respectively. Mean relative errors (MREs) for FDP and FCP are used to predict the fault behavior and the smaller value of MRE gives better predictive performance. MRE is expressed as follows

$$\begin{aligned} \frac{1}{2n} \sum _{i=1}^{n}\left( \bigg |\frac{m_d(t_i)-y_{d_i}}{y_{d_i}}\bigg | + \bigg |\frac{m_c(t_i)-y_{c_i}}{y_{c_i}}\bigg |\right) , \end{aligned}$$

where $m_d(t_i)$ and $m_c(t_i)$ are the estimated cumulative number of detected and corrected faults, and $y_{d_i}$ and $y_{c_i}$ are the cumulative number of detected and corrected faults at time $t_i$, respectively. Akaike information criterion (AIC) is defined through log-likelihood term and number of parameters in the model to estimate the relative amount of information lost. In regression model, AIC is calculated from sum squared error (SSE)^49,50^ and given as

$$\begin{aligned} AIC= n\;\ln \left( \frac{SSE}{n}\right) +2k, \end{aligned}$$

where *k* is number of parameter in the model.

### Predictive performance

In this subsection, we have estimated the parameters of all selected and proposed models. The least-square method is used to estimate the model’s parameters. All models’ performance is evaluated based on the goodness-of-fit criteria. From the model chosen, the G-O model is taken for FDP. Fault correction is associated with fault detection. FCP is modeled as a delayed FDP. The time delay ($\Delta t$) is the difference between fault detection and correction. FCP is modeled by taking different time delays with the G-O model. The different time delays the G-O model brings (model number 1 to 5) are constant delay, time-dependent delay, exponential delay, Normally distributed delay, and Gamma distributed delay.

#### Discussion of the FDP and FCP (DSI)

This subsection provides a detailed analysis of Tables 5 and 6, which present numerical estimations of the parameters used in the proposed Model. They compare its performance with other existing models using four key goodness-of-fit criteria: MSE, $R^2$, MRE, and AIC. A lower value of MSE, MRE, and AIC generally indicates a better-fitting model. However, $R^2$ follows a different rule: a higher $R^2$ value signifies a stronger correlation between the observed and predicted values, meaning the model explains more variance in the dataset. Thus, an ideal model should minimize MSE, MRE, and AIC while maximizing $R^2$. The GOF criteria in Table 6, the proposed model (referred to as model number 9) provides a superior fit compared to the other models under consideration.

**Fig. 4:**
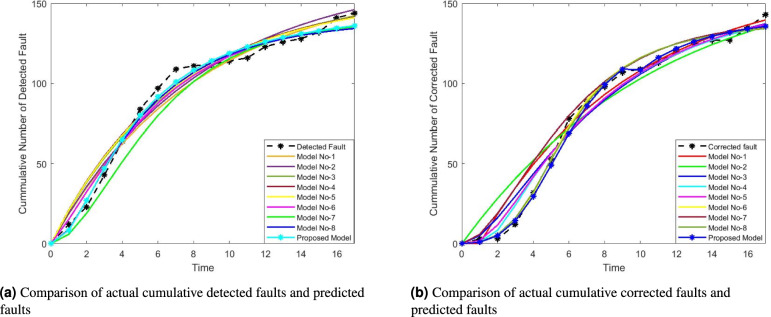
MVF of fault detection and correction of different models for DSI.

**Fig. 5:**
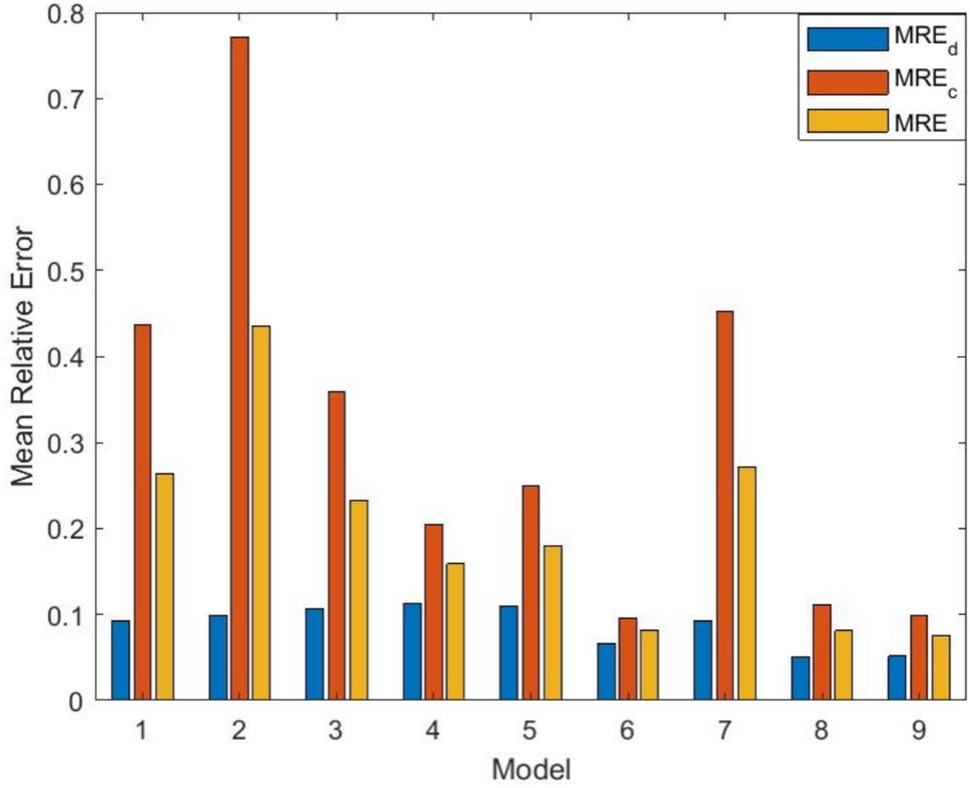
Comparison of prediction results for different models for DSI.

Here, specific numerical values for the GOF criteria are given to support the claim of the proposed model’s superior performance. The model achieves the lowest MSE, MRE, and AIC, indicating minimal error and better generalization capability. Additionally, its $R^2$ value is the highest, suggesting a strong alignment between the predicted and observed fault data. Figure 4a and b visually represent the MVF for fault detection and correction. These figures illustrate how well the proposed model tracks the actual fault detection and correction trends over time compared to existing models. Analyzing the figures shows that the fault detection and correction processes modeled by the proposed approach closely align with actual data. This observation reinforces the claim that the proposed model provides a better goodness-of-fit than the competing models. Further, it emphasizes that the proposed one achieves the lowest MSE among all models. A lower MSE indicates that the predicted values are closer to the actual values, signifying better predictive accuracy. MRE has been calculated and visualized in Fig. 5 to validate the model’s reliability further. MRE measures the average difference between predicted and actual values relative to the actual values, indicating the model’s relative accuracy. The conclusion restates that, based on the goodness-of-fit analysis and numerical evaluation, the proposed model is better suited for accurately modeling fault detection and correction. Additionally, the model’s ability to generalize well suggests that it can effectively predict future software failure trends, making it useful for real-world software reliability assessments.

**Table 5:** Estimated parameter values for data set DSI.

Model No	Estimated Parameters value
1	${a}=163.5541,\;{b}=0.12037,\;{c}=0.99994$
2	${a}=168.3642,\;{b}=0.119257,\;{c}=0.027895$
3	${a}=156.3445,\;{b}=0.140421,\;{c}=0.581124$
4	${a}=154.0903,\;{b}=0.146742,\;{\sigma }=0.7522,\;{\mu }=1.791183$
5	${a}=154.2259,\;{b}=0.145124,\;{\alpha }=2.583138,\;{\beta }=0.689097$
6	${a}=127.5765,\;{\alpha }=0.082116,\;{c}=0.125497,\;{r}=1.204778,\;{b}=27.87777,\;{\beta }=0.82659$
7	${a}=139.0387,\;{\alpha }=0.001,\;{r}=0.321904,\;{b}=1.204778,\;{\beta }=27.87984$
8	${a}=15.87525,\;{\alpha }=0.885984,\;{c}=5.894797,\;{r}=1.84221,\;{b}=15.13765,\;{\beta }=0.682886$
9	${p}=147.9287,\;{q}=5.554916,\;{\alpha }=0.966655,\;{b}=0.154198,\;{\beta }=15.82895,\;{r}=0.644238,\; {\gamma }=16.89578,\;{\sigma }=0.516214$

**Table 6:** Comparison results for different models for DSI.

Model No	$MSE_d$	$MSE_c$	*MSE*	$R_d^2$	$R_c^2$	$R^2$	$MRE_d$	$MRE_c$	*MRE*	*AIC*
1	55.75397	78.69555	67.22476	0.960045	0.955875	0.957960	0.092493	0.436317	0.264405	77.53613
2	58.05949	151.7182	104.8889	0.960666	0.888154	0.924410	0.098684	0.771911	0.435297	85.09932
3	50.66152	59.72248	55.19200	0.961992	0.966122	0.964057	0.107240	0.359117	0.233179	74.18391
4	52.23835	35.17967	43.70901	0.959998	0.981577	0.970787	0.112397	0.205127	0.158762	72.21842
5	50.84900	41.14912	45.99906	0.961014	0.978030	0.969522	0.110135	0.250338	0.180237	73.08656
6	37.64731	18.2954	27.97135	0.972123	0.991808	0.981965	0.06669	0.096225	0.081457	68.63008
7	85.01682	95.08205	90.04944	0.951775	0.946307	0.949041	0.093019	0.451526	0.272273	86.50610
8	26.57414	19.41566	22.99490	0.981818	0.991193	0.986505	0.051228	0.111113	0.081170	65.29963
9	21.726400	15.66265	**18.69452**	0.985451	0.992959	**0.989205**	0.051995	0.098886	**0.075441**	**64.85428**

Bold letters indicate that the proposed model gives better results than existing models for the respective goodness of fit criterion.

#### Discussion of the FDP and FCP (DSII)

Tables 7 and 8 present the estimated parameters of the proposed model alongside the comparison criteria values, calculated using the least square estimation (LSE) method. Table 8 serves as a performance benchmark, comparing the proposed model against existing models based on key goodness-of-fit criteria: MSE, $R^2$, MRE, and AIC. The results indicate that the proposed model (model number 9) achieves the lowest MSE (18.69452) and MRE (0.075441), suggesting a better fit to the dataset compared to the other models. Additionally, it attains the highest $R^2$ value (0.989205), demonstrating strong predictive accuracy. However, the AIC value of the proposed model (34.95918) is slightly higher than the smallest AIC value recorded among other models (31.46679). Despite this minor deviation, the overall performance of the proposed model remains superior, particularly in terms of minimizing prediction errors. Consequently, we conclude that model number 9 best fits dataset II compared to previously established models, such as those referenced in^51^.

**Fig. 6:**
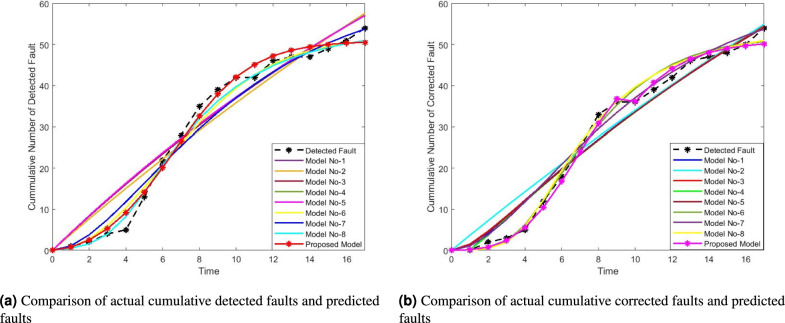
MVF of fault detection and correction of different models for DSII.

**Fig. 7:**
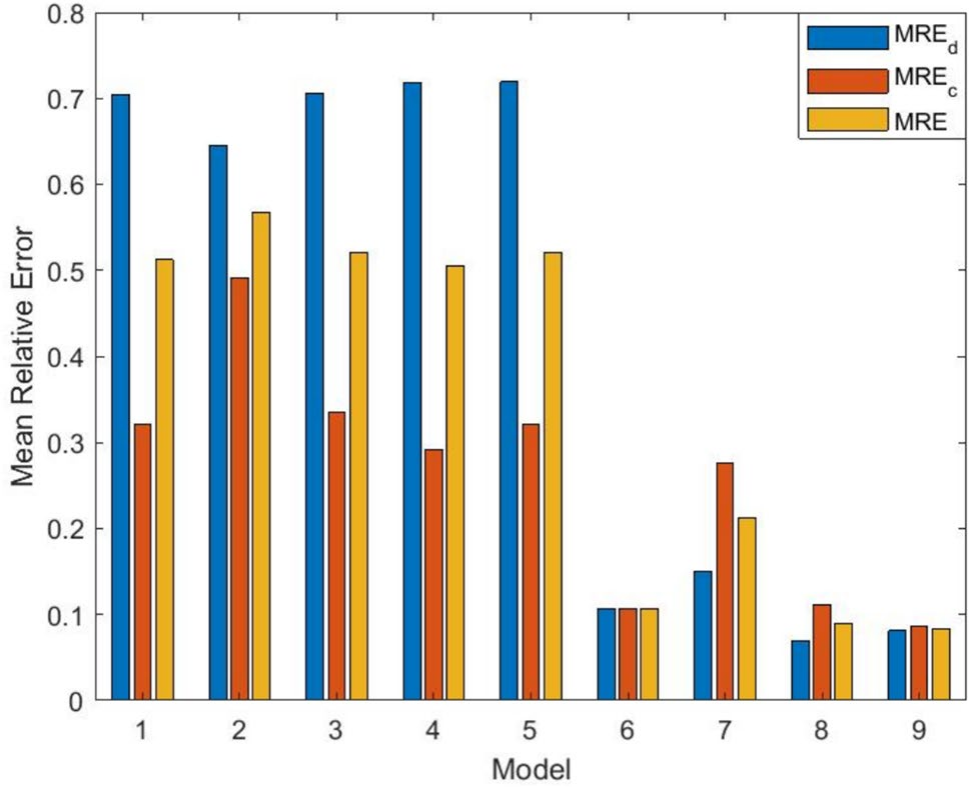
Comparison of prediction results for different models for DSII.

Further analysis is presented in Fig. 6a and b, which illustrate the mean value functions of the FDP and FCP for both the proposed and selected existing models. These visual representations reinforce the numerical findings, showing that the proposed model aligns more closely with the observed data. Additionally, the MRE for both FDP and FCP is calculated and graphically depicted in Fig. 7, further validating the model’s reliability. The results indicate that the proposed model accurately captures the fault detection and correction trends over time. Future research could focus on validating the proposed model with additional datasets and refining the comparison criteria to enhance its applicability across diverse software reliability scenarios.

**Table 7:** Estimated parameter values for data set DSII.

Model No	Estimated Parameters value
1	${a}=146.5184,\;{b}=0.029068,\;{c}=0.999998$
2	${a}=233.7391,\;{b}=0.016649,\;{c}=0.000907$
3	${a}=146.1045,\;{b}=0.029213,\;{c}=0.973239$
4	${a}=137.9221,\;{b}=0.031481,\;{\sigma }=0.199239,\;{\mu }=1.141654$
5	${a}=135.8696,\;{b}=0.032021,\;{\alpha }=1.227646,\;{\beta }=0.901345$
6	${a}=47.28639,\;{\alpha }=0.079457,\;{c}=0.01444,\;{r}=2.040396,\;{b}=28.13457,\;{\beta }=0.934013$
7	${a}=63.51191,\;{\alpha }=0.001,\;{r}=0.196226,\;{b}=1.204778,\;{\beta }=27.87984$
8	${a}=12.78458,\;{\alpha }=0.763122,\;{c}=39.98369,\;{r}=0.934761,\;{b}=18.09972,\;{\beta }=0.958901$
9	${p}=50.86054,\;{q}=0.056699,\;{\alpha }=1.011144,\;{b}=0.867000,\; {\beta }=7.381548,\; {r}=0.60325,\; {\gamma }=12.22316,\;{\sigma }=0.430075$

**Table 8:** Comparison results for different models for DSII.

Model No	$MSE_d$	$MSE_c$	*MSE*	$R_d^2$	$R_c^2$	$R^2$	$MRE_d$	$MRE_c$	*MRE*	*AIC*
1	24.14768	11.84240	17.99504	0.908761	0.957381	0.933071	0.704478	0.320755	0.512616	55.13163
2	23.97141	18.16542	21.06841	0.911707	0.926558	0.919132	0.644737	0.490800	0.567768	57.81217
3	24.20788	12.28588	18.24688	0.908749	0.954612	0.931681	0.706166	0.335887	0.521027	55.36790
4	24.49344	11.38663	17.94004	0.907511	0.959133	0.933322	0.718279	0.291497	0.504888	57.07959
5	24.46040	11.90931	18.18486	0.907139	0.956003	0.931571	0.719332	0.322111	0.520722	57.31002
6	5.574522	3.674572	4.624547	0.981919	0.989476	0.985698	0.107176	0.105971	0.106574	38.03343
7	10.66465	7.833082	9.248865	0.964108	0.973818	0.968963	0.150694	0.275678	0.213186	47.81651
8	2.933252	3.352293	3.142772	0.990987	0.990377	0.990682	0.069112	0.111226	0.090169	31.46679
9	3.896608	2.204027	**3.050318**	0.988359	0.993579	**0.990969**	0.081567	0.086433	**0.084000**	34.95918

Bold letters indicate that the proposed model gives better results than existing models for the respective goodness of fit criterion.

### Software reliability

Peng et al.^29^ defined software reliability as the probability of getting failure-free software within a time interval $(t, t+\delta t)$ such that the software releases at time *t* and the software doesn’t change in the operational phase. Thus, the software reliability function $R(\delta t\vert t)$ within $(t, t+\delta t)$ based on the proposed $m_d(t)$ is given by

13 $$\begin{aligned} R(\delta t\vert t)=\exp (-\lambda _d(t)\delta t), \end{aligned}$$

where $$\lambda _d(t)=\frac{dm_d(t)}{dt}$$.

## Optimal software release policy

The software’s quality depends upon the time spent on testing and the methodologies adopted during the testing phase. More faults will be removed if the software developer team spends more time in the testing phase. As a result, the software will become reliable, but the development cost of the software will be high. On the other hand, if the testing time of the software is too short, the price of the software is reduced, but there is a chance of increasing the operational cost of the software. Therefore, it is necessary to determine the optimal release time of the software. Many researchers have adopted cost criteria to assess the software’s optimal release time instead of the reliability concept^29,52,53^. Apart from the reliability criteria, we have discussed the optimal release time of the software based on the total cost spent during the testing and operational phase of the software. Therefore, we have considered the following cost model.

14 $$\begin{aligned} C(t)=C_0+C_1(t)m_d(t)+C_2(t)m_c(t)+C_3(t)(m_c(t_{lc})-m_c(t)), \end{aligned}$$

where $C_0$ is the setup cost for the software. We have considered the cost of fault detection to be linear to time, i.e., $C_1(t)=(C_1+\mu _1t)$, where $\mu _1$ is the unit charges for fault detection. So, the total cost for fault detection is $C_1(t)m_d(t)$. These faults are corrected after the detection of faults during the testing phase. Different factors affect the correction process while correcting the fault. Therefore, we have considered the cost of fixing unit fault as time-dependent, i.e., $C_2(t)=(C_2+\lambda _1t)$. The total cost for fixing the faults in the testing phase is $C_2(t)m_c(t)$. After fixing the faults, the software is released to the market, known as the operational phase. During this phase, fixing faults is a bit more challenging than the testing phase due to the absence of a software expert. Therefore, we have considered the testing and debugging cost of the software to be time-dependent in the operational phase as $C_3(t)(m_c(t_{lc})-m_c(t))=(C_3+\lambda _2(t_{lc}-t))(m_c(t_{lc})-m_c(t)).$ In the operational phase, the unit charges for fixing the faults are more significant than that of the testing phase, i.e., $\lambda _2>\lambda _1$. Concerning the proposed model, the estimated parameters $p=147.9287,\;q=5.554916,\;\alpha = 0.966655, \;b=0.154198, \;\beta =15.82895, \;r=0.644238, \; \gamma = 16.89578, \;\sigma =0.516214$ for DSI, is taken for software release time. We also assume $t_{lc}=100$, $C_0=\$100$, $C_1=\$15$, $C_2=\$15$, $C_3=\$25$, $\mu _1=0.15$, $\lambda _1=0.25$ and $\lambda _2=0.5$ for the software release.

**Fig. 8:**
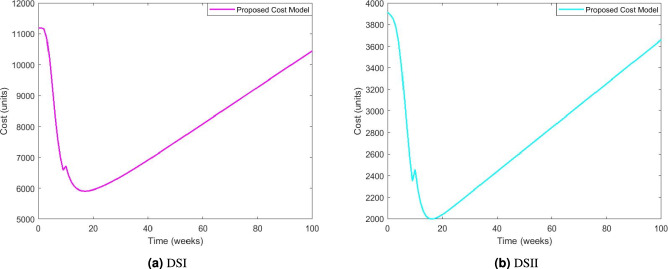
Predicted optimal release time for the proposed model.

Figure 8a presents the graphical representation of the cost function for DSI, which is used to determine the optimal release time of the software. The cost function is plotted against time to analyze the cost variation associated with different release periods. Initially, the cost function shows a decreasing trend, indicating that delaying the software release reduces the overall cost due to improved fault detection and correction. However, after a certain point, the cost function begins to rise, suggesting that further delaying the release increases costs, possibly due to extended testing efforts and delayed market entry. The graph shows that the cost function reaches its minimum value of $5915 at $$t=17.5$$ weeks. Beyond this point, any additional delay in the software release leads to increased costs. Therefore, based on the cost-minimization criteria, the optimal software release time is determined to be $$t^*=17.5$$ weeks, ensuring a balance between software quality and cost efficiency.

Similarly, the estimated parameters for the proposed model for DSII, i.e., $p=50.86054,\; q=0.056699, \;\alpha = 1.011144, \; b=0.867000, \;\beta =7.381548, \; r=0.603250, \;\gamma = 12.22316, \sigma =0.430075$, along with the various cost considerations, i.e., $t_{lc}=100\;\text {weeks}$, $C_0=\$100$, $C_1=\$15$, $C_2=\$15$, $C_3=\$25$, $\mu _1=0.15$, $\lambda _1=0.25$ and $\lambda _2=0.5$ associated with the software release have considered. To determine the optimal software release time, we have analyzed the cost function, which is graphically represented in Fig. 8b. The cost function for DSII follows a similar trend to that observed in DSI, where it initially decreases as the testing period progresses, reaching a minimum value before starting to rise again. This pattern suggests that delaying the software release initially helps in reducing costs due to enhanced fault detection and correction. Still, beyond a certain point, additional delays lead to increased costs, possibly due to prolonged testing expenses and delayed product deployment.

From the analysis, we observe that the cost function attains its minimum value of $2005.2 at $$t=17.5$$ weeks. This indicates that releasing the software at this point achieves the most cost-effective balance between testing efforts and market entry. Therefore, based on our cost-minimization approach, the optimal release time for DSII is $$t^*=17.5$$ weeks. A comparative summary of the optimal release time and corresponding costs for both DSI and DSII is provided in Table 9, highlighting the consistency of the proposed model in optimizing software release decisions across different datasets.

**Table 9:** Optimal release time and development cost for DSI and DSII.

Data sets	Optimal release time (weeks)	Optimal development cost ($)
DSI	17.5	5915.0
DSII	17.5	2005.2

## Conclusion

This study introduces a novel approach by incorporating multi-release concepts, fault dependency, and change points to enhance the modeling of FDP and FCP. The theoretical contributions include developing a general framework where fault correction occurs immediately after fault detection, considering fault dependency in FCP, and classifying detected faults into types I and II based on their origin. The introduction of the change point concept further refines the model by addressing variations in testing effort strategies required for different fault types. Additionally, using the LSE method, a systematic parameter estimation approach strengthens the theoretical foundation of SRGMs. Comparative analysis with existing models on real-world datasets demonstrates superior predictive performance, helping software teams make informed decisions about release timing. Furthermore, studying the optimal software release time through a cost model enables organizations to minimize costs while maintaining high reliability, making this approach highly applicable to industries aiming for cost-effective and dependable software deployment.

Several potential directions for future research are suggested. The proposed model can be extended into multi-release FDP and FCP, incorporating the uncertainty of the operating environment. The estimation of unknown parameters can be a interesting aspect to explore, and the maximum likelihood estimation method instead of the LSE method can be used to calculate the model’s parameters and accuracy by evaluating AIC, BIC, etc. The empirical data analysis used in this paper is to find the change point, but in the future, the software testing team is expected to know of this. The current model’s optimal release time is based on only a cost criterion that may be extended by incorporating reliability. We have not conducted a root cause analysis of the proposed model in this study. However, we acknowledge its importance and plan to explore it in future research.

## Data Availability

We confirm that the data supporting the findings of this study are cited and available within the article.

## References

[1] Goel, A. L. & Okumoto, K. Time-dependent error-detection rate model for software reliability and other performance measures. *IEEE Trans. Reliab.* **28**, 206–211 (1979).

[2] Pradhan, S. K., Kumar, A. & Kumar, V. An optimal software enhancement and customer growth model: a control-theoretic approach. *Int. J. Qual. Reliab. Manag.* **41**, 2333–2350 (2024).

[3] Yamada, S., Ohba, M. & Osaki, S. S-shaped reliability growth modeling for software error detection. *IEEE Trans. Reliab.* **32**, 475–484 (1983).

[4] Pham, H. Software reliability assessment: Imperfect debugging and multiple fault types in software development eg &g-raam-10737. *Idaho National Laboratory* (1993).

[5] Pradhan, S. K., Kumar, A. & Kumar, V. An effort allocation model for a three stage software reliability growth model. In *Predictive Analytics in System Reliability*, 263–282 (Springer, 2023).

[6] Pham, H. A new software reliability model with vtub-shaped fault-detection rate and the uncertainty of operating environments. *Optimization* **63**, 1481–1490 (2014).

[7] Pradhan, S. K., Kumar, A. & Kumar, V. Modelling reliability driven software release strategy considering testing effort with fault detection and correction processes: A control theoretic approach. *International Journal of Reliability, Quality and Safety Engineering* (2024).

[8] Pradhan, S. K., Kumar, A. & Kumar, V. A testing coverage based srgm subject to the uncertainty of the operating environment. In *Computer Sciences & Mathematics Forum*, vol. 7, 44 (MDPI, 2023).

[9] Chatterjee, S., Saha, D., Sharma, A. & Verma, Y. Reliability and optimal release time analysis for multi up-gradation software with imperfect debugging and varied testing coverage under the effect of random field environments. *Annals of Operations Research* 1–21 (2022).

[10] Pradhan, V., Dhar, J. & Kumar, A. Testing coverage-based software reliability growth model considering uncertainty of operating environment. *Systems Engineering* (2023).

[11] Pradhan, S. K., Kumar, A. & Kumar, V. An optimal resource allocation model considering two-phase software reliability growth model with testing effort and imperfect debugging. *Reliability: Theory & Applications* 241–255 (2021).

[12] Yang, C. et al. Cross-validation enhanced digital twin driven fault diagnosis methodology for minor faults of subsea production control system. *Mech. Syst. Signal Process.* **204**, 110813 (2023).

[13] Yang, C. et al. Digital twin-driven fault diagnosis method for composite faults by combining virtual and real data. *J. Ind. Inf. Integr.* **33**, 100469 (2023).

[14] Yang, C. et al. Fast and stable fault diagnosis method for composite fault of subsea production system. *Mech. Syst. Signal Process.* **226**, 112373 (2025).

[15] Ohba, M. Inflection S-shaped software reliability growth model. In *Stochastic Models in Reliability Theory*, 144–162 (Springer, 1984).

[16] Ohba, M. & Chou, X.-M. Does imperfect debugging affect software reliability growth? In *Proceedings of the 11th International Conference on Software Engineering*, 237–244 (1989).

[17] Pham, H. A software cost model with imperfect debugging, random life cycle and penalty cost. *Int. J. Syst. Sci.* **27**, 455–463 (1996).

[18] Pham, H. & Zhang, X. NHPP software reliability and cost models with testing coverage. *Eur. J. Oper. Res.* **145**, 443–454 (2003).

[19] Pillai, K. & Nair, V. S. A model for software development effort and cost estimation. *IEEE Trans. Software Eng.* **23**, 485–497 (1997).

[20] Kumar, V. & Pham, H. *Predictive Analytics in System Reliability* (Springer Nature, 2022).

[21] Yamada, S. & Osaki, S. Optimal software release policies with simultaneous cost and reliability requirements. *Eur. J. Oper. Res.* **31**, 46–51 (1987).

[22] Xie, M. & Hong, G. Software release time determination based on unbounded NHPP model. *Comput. Indus. Eng.* **37**, 165–168 (1999).

[23] Huang, C.-Y. & Lyu, M. R. Optimal release time for software systems considering cost, testing-effort, and test efficiency. *IEEE Trans. Reliab.* **54**, 583–591 (2005).

[24] Schneidewind, N. F. Analysis of error processes in computer software. In *Proceedings of the International Conference on Reliable Software*, 337–346 (1975).

[25] Xie, M., Hu, Q., Wu, Y. & Ng, S. H. A study of the modeling and analysis of software fault-detection and fault-correction processes. *Qual. Reliab. Eng. Int.* **23**, 459–470 (2007).

[26] Wu, Y., Hu, Q., Xie, M. & Ng, S. H. Modeling and analysis of software fault detection and correction process by considering time dependency. *IEEE Trans. Reliab.* **56**, 629–642 (2007).

[27] Huang, C.-Y. & Lin, C.-T. Software reliability analysis by considering fault dependency and debugging time lag. *IEEE Trans. Reliab.* **55**, 436–450 (2006).

[28] Lo, J.-H. & Huang, C.-Y. An integration of fault detection and correction processes in software reliability analysis. *J. Syst. Softw.* **79**, 1312–1323 (2006).

[29] Peng, R., Li, Y., Zhang, W. & Hu, Q. Testing effort dependent software reliability model for imperfect debugging process considering both detection and correction. *Reliab. Eng. Syst. Saf.* **126**, 37–43 (2014).

[30] Jia, L., Yang, B., Guo, S. & Park, D. H. Software reliability modeling considering fault correction process. *IEICE Trans. Inf. Syst.* **93**, 185–188 (2010).

[31] Chatterjee, S. & Shukla, A. Modeling and analysis of software fault detection and correction process through weibull-type fault reduction factor, change point and imperfect debugging. *Arab. J. Sci. Eng.* **41**, 5009–5025 (2016).

[32] Saraf, I. & Iqbal, J. Generalized software fault detection and correction modeling framework through imperfect debugging, error generation and change point. *Int. J. Inf. Technol.* **11**, 751–757 (2019).

[33] Pradhan, V., Dhar, J. & Kumar, A. Testing-effort based nhpp software reliability growth model with change-point approach. *Journal of Information Science & Engineering* **38** (2022).

[34] Yang, J., Liu, Y., Xie, M. & Zhao, M. Modeling and analysis of reliability of multi-release open source software incorporating both fault detection and correction processes. *J. Syst. Softw.* **115**, 102–110 (2016).

[35] Liu, Y., Li, D., Wang, L. & Hu, Q. A general modeling and analysis framework for software fault detection and correction process. *Softw. Test. Verif. Reliab.* **26**, 351–365 (2016).

[36] Zhu, M. & Pham, H. A multi-release software reliability modeling for open source software incorporating dependent fault detection process. *Ann. Oper. Res.* **269**, 773–790 (2018).

[37] Zhu, M. & Pham, H. A two-phase software reliability modeling involving with software fault dependency and imperfect fault removal. *Comput. Lang. Syst. Struct.* **53**, 27–42 (2018).

[38] Pham, H. & Zhang, X. An NHPP software reliability model and its comparison. *Int. J. Reliab. Qual. Saf. Eng.* **4**, 269–282 (1997).

[39] Leung, H. K. & White, L. A study of integration testing and software regression at the integration level. In *Proceedings. Conference on Software Maintenance 1990*, 290–301 (IEEE, 1990).

[40] Kapur, P., Pham, H., Anand, S. & Yadav, K. A unified approach for developing software reliability growth models in the presence of imperfect debugging and error generation. *IEEE Trans. Reliab.* **60**, 331–340 (2011).

[41] Song, K. Y., Chang, I. H. & Pham, H. An nhpp software reliability model with s-shaped growth curve subject to random operating environments and optimal release time. *Appl. Sci.* **7**, 1304 (2017).

[42] Li, Q. & Pham, H. Software reliability modeling incorporating fault detection and fault correction processes with testing coverage and fault amount dependency. *Mathematics* **10**, 60 (2021).

[43] Shu, Y., Wu, Z., Liu, H. & Yang, X. Software reliability modeling of fault detection and correction processes. In *2009 Annual Reliability and Maintainability Symposium*, 521–526 (IEEE, 2009).

[44] Musa, J. D., Iannino, A. & Okumoto, K. *Software Reliability: Measurement* (Application (McGraw-Hill Inc, Prediction, 1987).

[45] Shyur, H.-J. A stochastic software reliability model with imperfect-debugging and change-point. *J. Syst. Softw.* **66**, 135–141 (2003).

[46] Chang, Y.-P. Estimation of parameters for nonhomogeneous poisson process: Software reliability with change-point model. *Commun. Stat.-Simul. Comput.* **30**, 623–635 (2001).

[47] Chatterjee, S., Shukla, A. & Pham, H. Modeling and analysis of software fault detectability and removability with time variant fault exposure ratio, fault removal efficiency, and change point. *Proc. Inst. Mech. Eng. Part O* **233**, 246–256 (2019).

[48] Kapur, P., Gupta, A., Shatnawi, O. & Yadavalli, V. Testing effort control using flexible software reliability growth model with change point. *Int. J. Perform. Eng.* **2**, 245–262 (2006).

[49] Huang, Y.-S., Chiu, K.-C. & Chen, W.-M. A software reliability growth model for imperfect debugging. *J. Syst. Softw.* **188**, 111267 (2022).

[50] Saraf, I. & Iqbal, J. Generalized multi-release modelling of software reliability growth models from the perspective of two types of imperfect debugging and change point. *Qual. Reliab. Eng. Int.* **35**, 2358–2370 (2019).

[51] Zhu, M. & Pham, H. A software reliability model with time-dependent fault detection and fault removal. *Vietnam J. Comput. Sci.* **3**, 71–79 (2016).

[52] Lin, C.-T. & Huang, C.-Y. Enhancing and measuring the predictive capabilities of testing-effort dependent software reliability models. *J. Syst. Softw.* **81**, 1025–1038 (2008).

[53] Peng, R., Li, Y.-F., Zhang, J.-G. & Li, X. A risk-reduction approach for optimal software release time determination with the delay incurred cost. *Int. J. Syst. Sci.* **46**, 1628–1637 (2015).

